# Case Report: Right Bundle Brunch Block and QTc Prolongation in a Patient with COVID-19 Treated with Hydroxychloroquine

**DOI:** 10.4269/ajtmh.20-0376

**Published:** 2020-05-07

**Authors:** Rosmonaliza Asli, Muhammad Syafiq Abdullah, Pui Lin Chong, Dhiya Metussin, Riamiza Natalie Momin, Babu Ivan Mani, Vui Heng Chong

**Affiliations:** 1 Department of Medicine, RIPAS Hospital, Bandar Seri Begawan, Brunei Darussalam;; 2 Institute of Health Sciences, PAPRSB, Universiti Brunei Darussalam, Gadong, Brunei Darussalam;; 3 Department of Medicine, PMMPMHAMB Hospital, Tutong, Brunei Darussalam

## Abstract

Novel coronavirus disease (COVID-19) is a highly contagious disease caused by severe acute respiratory distress syndrome coronavirus-2 that has resulted in the current global pandemic. Currently, there is no available treatment proven to be effective against COVID-19, but multiple medications, including hydroxychloroquine (HCQ), are used off label. We report the case of a 60-year-old woman without any cardiac history who developed right bundle brunch block and critically prolonged corrected electrocardiographic QT interval (QTc 631 ms) after treatment for 3 days with HCQ, which resolved on discontinuation of the medication. This case highlights a significant and potentially life-threatening complication of HCQ use.

## INTRODUCTION

Novel coronavirus disease (COVID-19) is a highly contagious disease caused by severe acute respiratory distress syndrome coronavirus-2 (SARS-CoV-2) that was identified in December 2019 in China and is now a global pandemic.^1^ Currently, there is no proven effective treatment, and medications proposed to inhibit the virus life cycle such as hydroxychloroquine (HCQ), chloroquine, lopinavir/ritonavir, and remdesivir are used off label.^2–5^ These medications are widely used despite the lack of evidence for their efficacy and safety, and are often used in combination.

## CASE REPORT

A 60-year-old woman was admitted to the National Isolation Centre in Brunei after her nasopharyngeal and throat swabs tested positive (reverse transcriptase [RT]–PCR) for severe acute respiratory distress syndrome coronavirus-2 (SARS-CoV-2). She was among a group of infected travelers and was linked to a confirmed COVID-19 case through contact tracing. She had just returned from Indonesia 4 days before and developed symptoms (fever, dry cough, weakness, and dyspepsia) on returning. These symptoms had already improved when she was called for testing. Her comorbid conditions included hypertension, hyperlipidemia, and being overweight (31.1 kg/m^2^), but she had no known heart disease.

**Figure 1.**
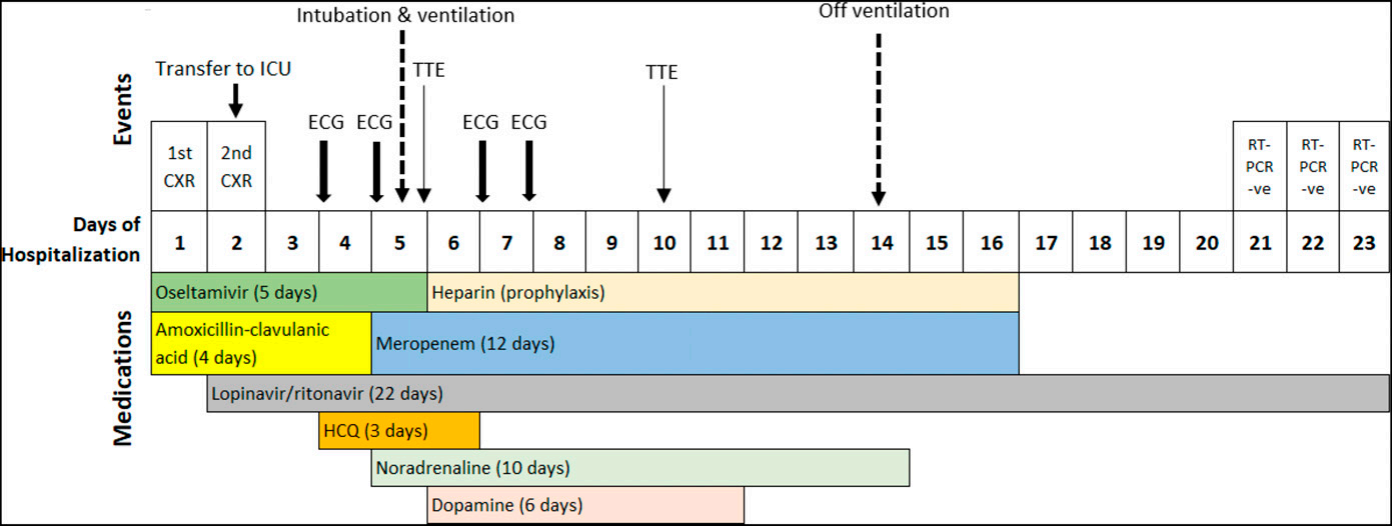
Timeline of treatment and events during patient’s hospalization (CXR = chest radiograph; ECG = electrocardiograph; ICU = intensive care unit; TTE: transthoracic echocardiogram).

Admission chest radiograph (CXR) was normal, and laboratory investigations revealed mildly elevated C-reactive protein, without lymphopenia (Table 1). She was empirically started on intravenous amoxicillin–clavulanic acid (1.2 g three times daily) and oseltamivir (75 mg twice daily). A repeat CXR on the second day of hospitalization showed bilateral lower zone opacities. As a result, she was transferred to the intensive care unit for close monitoring and was started on lopinavir 400 mg/ritonavir 100 mg (twice daily). As her condition did not improve, HCQ (400 mg stat dose followed by 200 mg twice daily) was initiated on the fourth day of hospitalization. An electrocardiograph (ECG) on hospital day 4 (before initiation of HCQ) was normal, with a corrected QT interval (QTc) of 397 ms. Repeat ECGs the following day remained normal (QTc 414 ms). The patient’s condition deteriorated, requiring intubation and ventilatory support on the fifth day of admission. Blood and urine cultures were negative. Sputum culture isolated *Pseudomonas aeruginosa* and *Serratia marcescens*, both sensitive to meropenem. Amoxicillin–clavulanic acid was discontinued, and meropenem (1,000 mg three times daily) was initiated. She was also started on inotropic support. The timeline of events and medications prescribed are shown in Figure 1 and laboratory investigations in Table 1.

**Table 1:** Laboratory results during the course of hospitalization

Day of hospitalization	1	5	6	7	8	9	10	19	22
Variable									
HB (g/dL) (11.5–15.9)	11	11.0	10.2	10.5	9.8	8.4	8.8	10.5	10.8
WCC (×10^9^) (4.2–12.6)	6.2	5.3	7.7	12.9	12.7	11.2	10.9	9.3	6.3
PLT (×10^9^) (174–430)	190	154	155	185	251	246	275	474	429
Lymphocyte (×10^9^)	1.2	0.9	0.9	0.4	1.5	1.3	1.4	2.1	1.7
Neutrophil (×10^9^)	4.6	4.2	6.5	12.3	10.3	9.6	9.1	6.6	3.8
Na^+^ (mmol/L) (136–144)	134	134	134	135	140	140	141	139	140
K^+^ (mmol/L) (3.5–5.1)	2.7	4.0	3.8	3.7	3.8	3.7	4.3	3.5	3.6
Cr (umol/L) (39–91)	73	80	76	75	76	65	67	57	70
Urea (mmo/L) (2.1–7.1)	3.3	2.8	2.6	2.8	6.0	7.1	90	5.6	3.5
Mg++ (mmol/dL(0.66–1.07)	–	0.68	0.69	0.87	0.96	0.99	0.96	–	–
Ca++ (mmol/L) (2.23–2.56)	–	2.13	2.17	2.14/2.25*	2.14/2.45*	2.33	2.47	–	–
Alb (g/L) (35–48)	36	31	–	29	–	–	20.4	29	32
ALT (U/L) (< 54)	41	32	–	46	–	–	60	60	62
GGT IU/L) (7–64)	18	16	–	29	–	–	57	51	44
ALP (U/L) (38–126)	54	39	–	36	–	–	59	60	58
Bilirubin (umol/L) (5.1–20.5)	9.4	20.3	–	105.6	–	–	50.5	23	30.2
T protein (g/L) (64–83)	70	67	–	64	–	–	60	63	66
CRP (mg/dL) (< 0.9)	1.1	8.0	–	–	–	–	36.8	–	0.8
Troponin I (ng/L) (< 30.0)	–	40.3/109.2	76.8 /115.6	66.3/46.8	–	–	–	–	–
Procalcitonin	–	–	–	0.43	–	–	–	–	–
D-dimer	–	–	–	506	–	–	–	3,189	2,149
Blood culture	–	–	–	–	–	–	–	–	–
Urine culture	–	–	–	–	–	–	-ve	–	–
Sputum culture	–	+ve†	–	–	–	–	–	–	–

HB = hemoglobin; WBC = white cell count; PLT = platelets.

* Calcium levels after intravenous calcium replacement.

† Sputum positive for *Pseudomonas aeruginosa* and *Serratia marcescens*.

**Figure 2.**
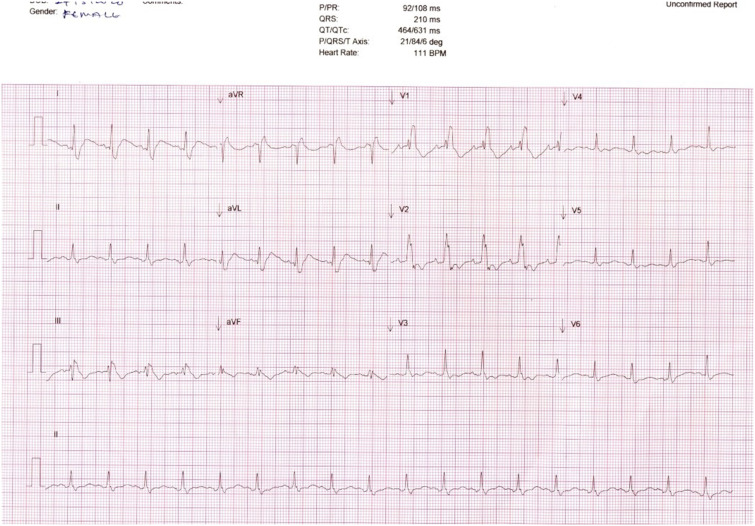
Electrocardiograph showing right bundle brunch block (RBBB) and prolonged corrected QT interval on day 4.

On the fifth day, serum troponin I was noted to be mildly elevated. Transthoracic echocardiogram (TTE) showed normal ejection fraction and no regional wall motion abnormalities. Myocarditis secondary to SARS-CoV-2 was considered. Serial monitoring twice daily showed fluctuation of troponin I. On the seventh day of hospitalization, repeat ECG before the morning dose of HCQ showed a new right bundle branch block (RBBB) and critically prolonged QTc (631 ms) (Figure 2). Hydroxychloroquine was discontinued after a cumulative dose of 1,400 mg. Blood investigations on that day showed normal serum Mg^2+^ and K^+^ but slightly low corrected Ca^2+^ (Table 1). This was corrected with calcium replacement. A repeat ECG performed 24 hours after the last dose of HCQ showed normalization of the QTc (433 ms).

On the tenth day of hospitalization, a repeat TTE showed normal cardiac function. She was eventually weaned off inotropes and was extubated on the 14th day of hospitalization. Investigations on the 19th day showed improvement of laboratory parameters apart from elevated D-dimer, and she was otherwise well and had no leg swellings. She was started on low molecular weight heparin. After three consecutive negative RT-PCR results for SARS-CoV-2, she was transferred on the 23rd day of hospitalization to a tertiary hospital, where a computed tomography pulmonary angiogram showed scattered ground-glass opacities consistent with COVID-19 and a small pulmonary embolism on the right. She was started on dabigatran 150 mg twice daily (planned 3 months of treatment) and remained well on follow-up.

## DISCUSSION

Corrected QT interval prolongation is dangerous and can be associated with torsade de pointes, a life-threatening arrhythmia. Our patient developed RBBB and critically prolonged QTc (QTc > 500 ms) after 3 days of HCQ at a cumulative dose of 1,400 mg. A systematic review of chronic use of chloroquine and HCQ in rheumatic conditions reported cardiac side effects to be common. Among patients who were treated with HCQ who experienced cardiac toxicity (*n* = 50, median duration of use 8 years [range 10 days to 30 years], and cumulative dose of 1,235 g [range 1.9 g–4,380 g]), the study reported bundle branch block (26%), atrioventricular block (24%), and first- or second-degree heart block (4%).^6^ Other cardiac adverse effects of HCQ included ventricular hypertrophy (32%), ventricular hypokinesia (16%), heart failure (ejection fraction < 40% in 52.9%), and valvular dysfunction (8%), especially with high cumulative doses.^6^ Other adverse effects of HCQ include gastrointestinal, ophthalmic, neurological, musculoskeletal, psychiatric, metabolic, and dermatological abnormalities.^7^

Patients with COVID-19 who require hospitalization are at risk for complications including electrolyte derangements, which are risk factors for QTc prolongation.^8^ Our patient had several risk factors for conduction abnormalities: administration of HCQ, lopinavir/ritonavir, and inotropes, and hypocalcemia. Lopinavir/ritonavir is also associated with the prolongation of QTc, but the ECG after starting this medication was normal. Among the electrolytes associated with the prolongation of QTc, only calcium was slightly low. Inotropes were started 2 days (noradrenaline) and one (dopamine) day before the detection of conduction abnormalities. Unfortunately, we did not obtain an ECG on the third day of HCQ therapy. We considered the possibility of myocarditis. The elevated troponin coincided with the period leading up to conduction abnormalities and peaked several days later, before decreasing. A repeat TTE was normal. We did not perform further investigations for myocarditis, as our patient remained stable. It is possible the other factors discussed contributed to the development of conduction abnormalities, but the resolution of ECGs changes after discontinuation of HCQ suggested a causal relationship between HCQ and these abnormalities.

Given the lack of proven therapies for COVID-19, the continued use of HCQ is likely. HCQ and chloroquine have also been used as prophylaxis for COVID-19.^7,9^ With such widespread use, complications can be expected. Combination therapy of chloroquine and azithromycin, both medications associated with QTc prolongation drugs, has recently been advocated.^4^ Our case highlights that significant and life-threatening conduction abnormalities can occur with the use of HCQ. Therefore, clinicians should exercise caution and assess cardiac risk if considering HCQ treatment for COVID-19.
